# The Effects of *Laurencia caspica* Algae Extract on Hemato-Immunological Parameters, Antioxidant Defense, and Resistance against *Streptococcus agalactiae* in Nile tilapia (*Oreochromis niloticus*)

**DOI:** 10.1155/2023/8882736

**Published:** 2023-07-04

**Authors:** Majid Khanzadeh, Babak Beikzadeh, Seyed Hossein Hoseinifar

**Affiliations:** ^1^Animal Biological Product Research Group, Academic Center for Education, Culture and Research (ACECR), Tehran, Tehran Organization, Iran; ^2^Faculty of Fisheries and Environmental Sciences, Gorgan University of Agricultural Sciences and Natural Resources, Gorgan, Iran; ^3^Department of Cell and Molecular Biology & Microbiology, Faculty of Biological Sciences and Technology, University of Isfahan, Isfahan, Iran

## Abstract

Natural immune stimulants are among the most effective chemicals for boosting immunity and fish welfare. This study aims to investigate the effects of red macroalgae extract (*Laurencia caspica*) on hematological, immunological, antioxidant, biochemical, and disease resistance against *S. agalactiae* in Nile tilapia for 50 days. For this purpose, fishes were assigned to four dietary treatments group in which the base meal was supplemented with 0.5%, 1%, and 2% of *L. caspica* extract. On days 25 and 50 of the experiment, samples were taken to investigate the hematological, immunological, biochemical, and antioxidant parameters. The white blood cells (WBCs), hemoglobin, and neutrophils significantly increased after 50 days of feeding with the *L. caspica* extract, but until the 25th day, no significant difference was observed among the treatments except for hemoglobin. Immunological parameters (including Immunoglobulin M [IgM] and complement 3 [C3]) were significantly higher in treated groups compared to control both 25 days and 50 days posttreatment. However, on the 25th day, no significant difference was noticed between treatments and control in the case of lysozyme activity. Alkaline phosphatase (ALP) and alanine aminotransferase (ALT) considerably increased in comparison to the control group on the 50th day, but no significant difference was observed on the 25th day. In addition, feeding with *L. caspica* significantly increased the antioxidant enzyme activities on the 25th day (*L. caspica* 1% and 2% in peroxidase [POD] and superoxide dismutase [SOD] in all groups) and 50th day (catalase [CAT], SOD and *L. caspica* 1% and 2% in POD) in the spleen. The survival rate of fish challenged with *Streptococcus agalactiae* was considerably greater than the control group. Finally, it can be concluded that *L. caspica* extract is an immunological stimulant that induces fish resistance to *S. agalactiae*.

## 1. Introduction

As the world population rises, food availability is the most critical factor affecting human life. Aquatic animal production and consumption are the most significant answers to this problem [1]. Aquaculture is a great source of healthy and quality proteins for humans with all types of tastes [2]. However, the spread of infectious diseases is a major problem that threatens this progress [3]. Bacterial diseases are the most common challenge that threatens the aquaculture industry [4]. Antibiotic therapy is a major method to control bacterial diseases; however, as a result of bacterial resistance to antibiotics, antibiotic usage has been restricted. In addition, antibiotics are also responsible for killing beneficial bacteria [5, 6]. The potential alternative to antibiotics is the use of additives as immunostimulants, which improves disease resistance in aquatic animals by strengthening the defense mechanisms inherent in aquaculture [7, 8].

On the other hand, the increased demand for aquaculture leads to an intensification of stress factors and susceptibility to some diseases in Nile tilapia fish [9]. For instance, *Streptococcus agalactiae* is regarded as the most significant pathogen in Nile tilapia breeding [10]. *S. agalactiae* and *Streptococcus iniae* are the main bacterial tilapia pathogens that may cause severe symptoms with more than 50% mortality [11]. Although vaccination is the most significant method for disease prevention [12], antibiotics are the first choice in aquaculture.

Immune-boosting herbs are more reliable and safe medications for the treatment of diseases. The main group of bioactive compounds abundantly found in red seaweeds are agar, alginates, and carrageenan, which are used in various ways such as human nutrition, animal feed, or manure. Extracted compounds from seaweeds have antimicrobial, antioxidant, and therapeutic activities; they have good potential to be used as supplements in food formulas [13]. Red algae *L*. *caspica* is a marine species that belongs to the category of multinucleated cell algae. This algae is a rich source of vitamins A, B, C, E, and vitamin K, as well as vital minerals such as magnesium, calcium, copper, potassium, selenium, zinc, iodine, and iron [14]. Recent studies revealed that red algae, in addition to low fat, contains all of the essential amino acids and omega-3 fatty acids for the body [14].

Since ancient times, Nile tilapia (*Oreochromis niloticus*) (Linnaeus, 1758) is the most widely cultured aquatic species [15]. Due to its rapid maturation and complicated immune system, this species is also considered as a good choice for aquaculture development study [16, 17]. Moreover, the market for Nile tilapia has grown to meet the protein requirement of the middle class due to its availability, pleasant flavor, and cost-effectiveness [18]. Currently, more than 140 countries in the world are producing and raising tilapia fish [19]. The global harvest of farmed tilapia has exceeded 6 million tonnes (MT), placing tilapia second only to carp as the most widely consumed freshwater fish in the world [20].

Previous research showed that algal extract has a beneficial impact on immunological and blood markers as well as bacterial resistance. However, no research has been done on *Laurencia caspica* effects. Considering the beneficial characteristics of macroalgae and the ubiquity of *L. caspica* in the Caspian Sea, the purpose of this study is to investigate the impact of this red algae (*L. caspica*) on the hematological, immunological, and antioxidant factors, and the relative survival percentage of Nile tilapia in response to *S. agalactiae*.

## 2. Material and Methods

### 2.1. Algae Collection and Extraction

*L. caspica* was collected in Mazandaran, Iran, on the southern Caspian Sea beaches. It was washed, let to air dry for 48 hr at room temperature, and then stored in a cold, dry location until extraction. The hydroethanolic extraction by maceration technique [21] was performed using a rotating apparatus: 20 g of dry algae powder was combined with 300 ml of water and 70% ethanol (1 : 15 v/v) for 72 hr, and the solvent was separated by rotary evaporation. The extract was freeze-dried and stored at 4°C until use [22].

### 2.2. Diet Preparation and In Vivo Experimental Design

Nile tilapia (600 pieces) with an average weight of 100 ± 5 g were obtained from a farm in Bafgh, Yazd, Iran, and then housed in tanks. After 14 days of acclimation, the fishes were randomly distributed into four treatment groups and three replicate groups in 12 tanks (each tank containing 50 fishes). The control group diet (without *L. caspica* extract) and treatment groups with 0.5%, 1%, and 2% of *L. caspica* extract were fed for 50 days [23]. To prepare the diet for treatment groups, the *L. caspica* extract was added to the commercial feed (21 Beyza Mill Co., Shiraz, Iran) containing 37% crude protein, 10% crude fat, less than 10% moisture, and 4,000 kcal/kg digestible energy as the base diet. The *L. caspica* extract was dissolved in 30 ml of distillate water before being combined with feeds. Fifty milliliters per kilogram of a 3% gelatin solution were added to the diet to prevent the extracted active ingredients from being lost in the water. Fishes were fed daily at a 2% body weight ratio.

### 2.3. Blood Samples and Hematological Parameters

Blood collection intervals were divided into 25-day and 50-day phases. Three fishes were collected from each replication, for a total of nine fishes per treatment. The blood was collected from the caudal vein of the fish using nonheparinized 2 ml syringes. To isolate serum and plasma, the blood samples were divided into 1 ml tubes with no anticoagulant and 1 ml tubes with anticoagulant heparin, respectively. The anticoagulant-free samples were centrifuged for 15 min at 3,000 rpm, and the serum was kept at −20°C until further examination. Hemoglobin was measured according to Telli et al. [24]. First, the solution was produced in DROBKINS by combining 15 ml of the reagent with 500 ml of distilled water. Then we put 10 *μ*l of blood and 2.5 ml of the solution into the test tube (ratio 1 : 250). Then, a spectrophotometer was used to detect the absorbance at 546 nm. The hemoglobin concentration in grams per deciliter is obtained by multiplying the value by 36.9. Mean corpuscular volume (MCV), mean corpuscular hemoglobin (MCH), and mean corpuscular hemoglobin concentration (MCHC) were all calculated following the method described by Telli et al. [24]. Total red blood cell (RBC) and white blood cell (WBC) counts were conducted using a Neubauer hemocytometer slide and a 1 : 200 dilution of blood with (Natt–Herrick) dilution solution [24]. The blood sample was processed and stained with Giemsa dye for differential counting of WBCs [25].

### 2.4. Antioxidant Enzymes Assay

To investigate antioxidant enzyme activity, the spleen (1 g) was homogenized in 4 ml of potassium phosphate buffer (50 mM, pH = 7) containing ethylenediaminetetraacetic acid and polyvinylpyrrolidone (PVP) for the antioxidant enzymes test. The mixture was centrifuged at 10,000 rpm for 10 min at 4°C, and the supernatant was frozen at −20°C for further examination. According to Beauchamp and Fridovich [26], superoxide dismutase (SOD) activity was assessed by the decreased absorption rate of Nitroblue tetrazolium (NBT) in the presence of SOD. Catalase (CAT) activity was determined based on the change in 240 nm absorbance generated by hydrogen peroxide in 1 min, as reported by Zengin and Yilmaz [27]. Peroxidase activity was estimated based on Chance and Maehly measured at 470 nm by spectrophotometer [28].

### 2.5. Serum Biochemical Parameters

Following the methodology described by Hoseinifar et al. [29] alkaline phosphatase (ALP), aspartate aminotransferase (AST), and alanine aminotransferase (ALT), levels in serum were measured using commercial kits from Pars Azmoun in Tehran, Iran.

### 2.6. Lysozyme Assay

The enzyme-linked immunosorbent assay (ELISA) method was used to measure lysozyme levels as described by Ross et al. [30] First, 9 mg of *Micrococcus luteus* wall was dissolved in 30 ml of phosphate buffer to test lysozyme. Then, 10 *μ*l of serum was put into each well of the microplate, followed by 90 *μ*L of wall suspension. The ELISA results were determined at a wavelength of 450 nm within 10 min [30].

### 2.7. Immunoglobulin M (IgM) Assay

Serum IgM levels in each group were measured on days 25 and 50 by the ELISA method described by Cuesta et al. [31]. Blood samples were collected from the caudal vein of treatment and control fish on days 25 and 50. (*n* = 9 per group) and centrifuged at 3,000 rpm for 15 min to separate the serum. Briefly, 96-well plates were coated serially with 100 ml of diluted serum samples (1 : 200) and incubated at 4°C overnight. Blocking the wells with 5% skim milk was performed for two hours at room temperature. After washing, 1 : 2000 dilutions of 100 ml of mouse anti-Nile tilapia IgM polyclonal antibody were added to each well and incubated at 37°C for 37 hr. A secondary rabbit antimouse antibody solution containing 100 microliters of horseradish peroxidase (HRP) (1 : 2,000) was added, and the mixture was incubated at 37°C for 1.5 hr. Finally, O-phenylenediamine dihydrochloride (OPD) was utilized and the reaction was terminated with 25 ml of 2 M H_2_SO_4_ in each well, and the absorbance was measured at 490 nm [31].

### 2.8. C3 Assay

The amount of C3 proteins were measured using commercial kits (Pars Azmoon, Tehran, Iran) according to the He et al. [32] method.

### 2.9. S. Agalactiae Challenge Test

*S. agalactiae* obtained from tilapia kidneys from the laboratory of Tehran Aquatic Clinic (Tehran, Iran) was used as a virulent strain in fish. Fifty days after feeding, 20 fishes from each experimental group received an intraperitoneal injection (IP) of 0.1 ml of live *S. agalactiae* (1 × 10^8^ CFU ml^−1^). The mortality of fish was observed for 14 days (until day 64 of the experiment), and postinfection survival was calculated using the following formula [33].

(1) $$\begin{array}{c} \text{Relative percentage survival }\left(RPS\%\right)=\left[\text{Number of surviving fish after challenge }/\text{Number of fish injected with bacteria}\right]\times 100 \end{array}$$

### 2.10. Statistical Analysis

The GraphPad PRISM software (version 9) was used to analyze data. First, the standard deviation (SD) of the data was checked for normality and homogeneity using the Leven statistic test. Then, two-way analysis of variance and Tukey analysis of variance was performed to compare groups over time. The data are presented as ± SD.

## 3. Results

### 3.1. Hematological Parameters

On days 25 and 50, the RBC, hematocrit, MCV, MCH, MCHC, lymphocyte, monocyte, and blood eosinophil characteristics were not statistically different between treatment groups (*P* > 0.05). On day 50, the number of WBCs in the treatment groups with *L. caspica* extract 1% and *L. caspica* extract 2% was significantly higher than in the control group (*P* < 0.05), but on day 25 there was no significant difference (*P* > 0.05). Compared to the control group, the quantity of hemoglobin in *L. caspica* was 1% and *L. caspica* 2% on the 25th day, and *L. caspica* 1% on the 50th day of sampling increased significantly (*P* < 0.05). On the 50th day of sampling, the proportion of neutrophils in group *L. caspica* 2% was significantly higher than in the control group (Table 1, *P* < 0.05). The main effect of time between days 25 and 50 was a significant increase in WBC count. Thus, by increasing the day, the number of WBCs has increased significantly (*P* < 0.05). The main effect of time on RBC count (*L. caspica* 1% and 2%), hemoglobin (*L. caspica* 1% and 2%), percent of hematocrit (*L. caspica* 1% and 2%), MCV (*L. caspica* 1%), neutrophils (*L. caspica* 0.5%), lymphocytes (*L. caspica* 0.5%), monocytes (*L. caspica* 2%), and eosinophils (*L. caspica* 2%) had a significant increase (Table 1, *P* < 0.05). Regarding the interaction between time and treatment, there was a significant increase in factors such as WBC, RBC, hemoglobin, and hematocrit (Table 1, *P* < 0.05), but no significant effect was obtained in the case of other parameters (Table 1, *P* > 0.05).

### 3.2. Lysozyme

**Figure 1:**
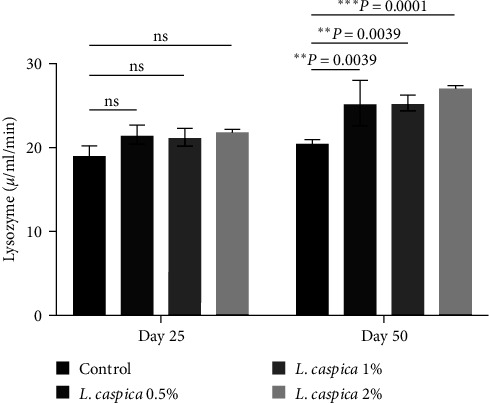
Lysozyme activity of Nile tilapia treated with various doses of extract of *L. caspica*. Each bar represents mean ± SD. There were significant differences among treatments (*P* ≤ 0.05). ns, not significant.

On day 50, the serum lysozyme was significantly increased in fish fed with 0.5%, 1%, and 2% *L. caspica* extract compared to the control group (Table 2, Figure 1, *P* < 0.05). Compared to the 25th day, the main impact of time on the 50th day was a considerable rise in lysozyme (*L. caspica* 0.5%, 1%, and 2%) (Table 2, Figure 1, *P* < 0.05). Regarding the interaction effect between time and treatment, no significant difference was observed in the case of serum lysozyme activity (Table 2, *P* = 0.1074).

### 3.3. IgM

**Figure 2:**
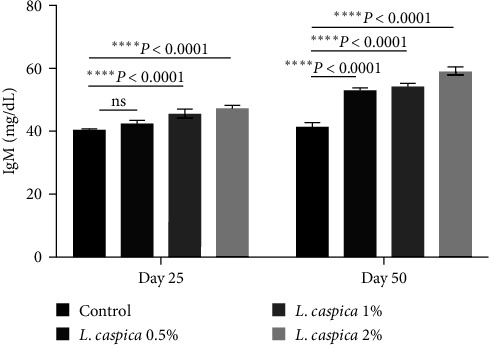
Total IgM activity of Nile tilapia treated with various doses of extract of *L. caspica*. Each bar represents mean ± SD. There were significant differences among treatments (*P* < 0.05). ns, not significant.

On day 25 and day 50, *L. caspica* extracts 0.5%, 1%, and 2% IgM levels, respectively, were significantly higher than those of the control group (Table 2, Figure 2). The primary effect of time on the 50th day was a significant increase in IgM level compared to the 25th day (Table 2, Figure 2). The study of the interaction effect between time and treatment revealed a significant difference in the case of serum IgM level (Table 2, *P* < 0.0001).

### 3.4. C3

**Figure 3:**
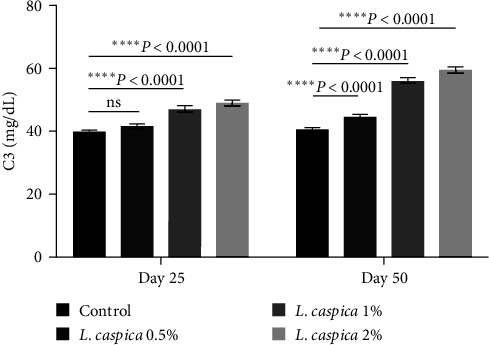
C3 activity of Nile tilapia treated with various doses of extract of *L. caspica*. Each bar represents mean ± SD. There were significant differences among the treatments (*P* < 0.05). ns, not significant.

The C3 protein levels in *L. caspica* extract at 1%, and 2% on the 25th day and *L. caspica* at 0.5%, 1%, and 2% on the 50th day were significantly different from those of the control group (Table 2, Figure 3, *P* < 0.05). The main impact of time on the 50th day relative to the 25th day was a considerable rise in C3 (*L. caspica* 0.5%, 1%, and 2%), (Table 2, Figure 3, *P* < 0.05). Evaluation of the interaction effect between time and treatment showed a significant difference in the case of the C3 level (Table 2, *P* < 0.0001).

### 3.5. Biochemical Parameters

ALP levels at 0.5%, 1%, and 2% of *L. caspica* extract on day 50 were significantly higher than the control group, whereas ALT levels at 2% of *L. caspica* extract were significantly different from the control group (*P* < 0.05). However, on different sampling days, there was no significant difference in AST levels across groups (Table 3, *P* > 0.05). A significant increase in ALP (*L. caspica* 1% and 2%) and ALT (*L. caspica* 1% and 2%) was the major effect of time on the 50th day compared to the 25th day (Table 3, *P* < 0.05). Regarding the interaction effect of time and treatment, a significant difference was noticed in cases of ALP and ALT (Table 3, *P* < 0.05), but no significant difference was noticed in the case of AST (Table 3, *P* = 0.3778).

### 3.6. Antioxidant Activity

CAT on the 50th day in 0.5%, 1%, and 2% of *L. caspica* extract and peroxidase (POD) on the days 25 and 50 at 1% and 2%, of *L. caspica* extract was substantially increased from the control group (*P* < 0.05). On the 25th day, the SOD at 0.5%, 1%, and 2% of *L. caspica* extract, and on day 50, the SOD at 0.5%, 1%, and 2% of *L. caspica* extract substantially increase from the control group (Table 4, *P* < 0.05). The main impact of time was a considerable rise in CAT (*L. caspica* 0.5%, 1%, and 2%) and POD (*L. caspica* 2%) on the 50th day compared to the 25th day (Table 4, *P* < 0.05). For SOD, the major impact of time was not significant (Table 4, *P* > 0.05). The study of the interaction effect of time and treatment revealed no significant difference in cases of serum SOD and POD levels (Table 4, *P* > 0.05). However, there was a significant effect on the serum CAT level (Table 4, *P* < 0.0001).

### 3.7. Survival Postchallenge

**Figure 4:**
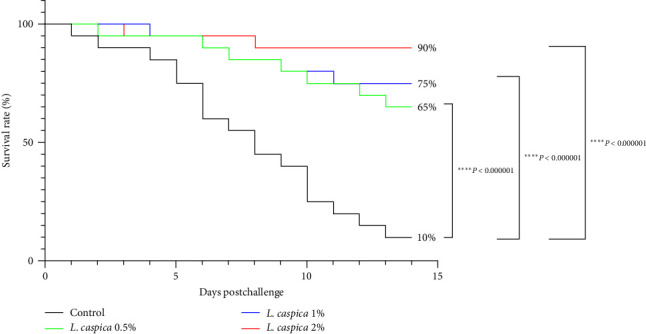
Kaplan–Meier estimate of cumulative survival rate for 14 days after the treated Nile tilapia challenged with *S. agalactiae*.

After 14 days of the Streptococcus challenge, survival rates of fish treated with *L. caspica* extract 0.5%, 1%, and 2% were considerably higher than the control group (*P* < 0.05). The *L. caspica* extract 2% had the best survival rate (90%), whereas only 10% of the fish in the control group survived (Figure 4).

## 4. Discussion

In this study, fish fed diets incorporated with algae extract had significantly increased WBC, hemoglobin, and neutrophil counts on different sampling days (days 25 and 50) compared to the control group. In a study conducted by Adel et al. [34] dietary *Spirulina platensis* (5%–10%) enhanced RBC, WBC, Hb, and neutrophils in the blood of sturgeon, which is consistent with our findings. In another study, *Sargassum angustifolium* algae extract enhanced the number of RBCs, WBCs, hematocrit, and hemoglobin in rainbow trout (*Oncohrynchus mykiss*) blood [35]. There is a possible explanation for the increase in blood hemoglobin, which causes the protective effect of polyphenols against hydrogen peroxide caused by oxidation in RBCs. Additionally, the increase in blood hemoglobin may have been influenced by the antioxidants in algal extract, which reduced hemolysis by peroxidation of lipids in RBC membranes [36]. Macroalgae contain a lot of polyphenols, which are crucial for avoiding oxidation [37]. One of the most important cells that might cause nonspecific immunological reactions in fish is the WBC, [38]. In naturally occurring and experimental diseases, as well as, using different vaccinations and immunological stimulants, a rise in WBCs has been recorded [39]. After 30 days of feeding *Ulva clathrate* algae extract by Nile tilapia, a substantial rise in WBC count was found, which is consistent with the findings of this research [40]. In addition, *S. angustifolium* algae extract enhanced the quantity of WBCs in rainbow trout (*O. mykiss*), [41]. The increase in neutrophils is most likely due to the presence of carotenoids in the algae, which stimulate neutrophil, macrophage, and lysozyme formation in the blood, increase phagocytic activity, and so enhance nonspecific immunity [42]. *Labeo rohita* fish showed improved blood neutrophil activity after being fed *Chaetomorpha antennina* algae extract [43]. Red algae (*L. caspica*) increased blood parameters such as RBCs, WBCs, hemoglobin, and hematocrit compared to the control group in goldfish [44]. The mixture of three algae (*Ulva lactuca*, *Jania rubens*, and *Pterocladia capillacea*) had a significant increase in the amount of (RBCs, hemoglobin, haematocrit, and WBCs) compared to the control group in catfish [45].

Fish have a general defense mechanism called lysozyme, which is a cationic protein with a low molecular weight [46]. Lysozyme levels were greater in the 2% *L. caspica* extract-treated fish on day 50 compared to the control group. An increase in lysozyme is usually accompanied by an increase in WBCs and eosinophilic activity in fish tissues [47] and reported in Black Sea bream (*Acanthopagrus schlegelii*), [48], common carp (*Cyprinus carpio*) [49], and rainbow trout (*O. mykiss*) [50]. The findings of the current investigation revealed that fish given 0.5%, 1%, and 2% supplements on various days had significantly higher IgM and C3 protein levels than the control group. Furthermore, fish fed 0.5%, 1%, and 2% *L. caspica* extract had significantly IgM and C3 levels than the control group, as previously observed in rainbow trout [50], Asian seabass (*Lates calcarifer*) [51], and Nile tilapia [52]. That fish fed complementary diets had greater immunity levels than fish on the control diet. Additionally, fish fed an algal extract-supplemented diet may have better digestive enzymes, which may enhance intestinal microbiota and subsequently stimulate the immune response [53]. Algae have phytochemical compounds that have antioxidant activity. Elevation of these compounds may increase blood lysozyme levels. Ashour et al. [54] reported an increase in serum lysozyme levels after feeding with algal extract in Nile tilapia fish.

Liver enzymes are often considered for the detection of fish infections and tissue damage. These enzymes' increased in extracellular fluid and serum as a sign of mild cell injury [55]. According to our results, fish fed *L. caspica* 2% algae extract exhibited a substantial rise in ALP and ALT factors on the 50th day compared to the control group. However, on several days, there was no significant difference in AST level across groups. Blood enzymes AST and ALT play a crucial role in cellular nitrogen metabolism, amino acid oxidation, and liver gluconeogenesis, and they may be used to check for toxic effects that cause liver damage or dysfunction [56]. ALP has antibacterial properties due to its hydrolytic activity. Therefore, an increase in ALP value indicates an improvement in immune status. This was also supported by Masoomi-Feshani and Vazirzadeh [57], who reported increased serum ALP in rainbow trout (*O. mykiss*) fed with probiotics for 30 days, as a sign of increased immunity in fish. Hoseinifar et al. [58] reported an increase of ALP in zebrafish using Gracilaria gracilis algae. It seems that a rise in the activity of these enzymes in the plasma of fish treated with *L. caspica* occurred because of liver tissue injury [59]. Improved ALT and AST enzyme functions are documented in Nile tilapia-fed spirulina algae [60], which is consistent with the current findings.

Antioxidant enzymes reveal the health of the body's antioxidant system, indicating the capacity to digest oxygen-free radicals and protect fish tissue from oxidative damage [61]. SOD and CAT enzymes are the first line of defense against oxidative stress and are often utilized as indicators of reactive oxygen species (ROS) generation [62, 63]. According to the findings, fish-fed *L. caspica* algae extract had significantly higher CAT, POD, and SOD factors on various days than the control group. Several mechanisms may be linked to these beneficial effects: (1) Natural antioxidants have a high potential to scavenge free radicals from the internal antioxidant system, (2) lipid peroxidation inhibition, and (3) reactive pure oxygen species reduce oxidative stress, protect cells, and other natural substances from harm [64]. In accordance with our findings, fucoidan (extracted from *Laminaria japonica* algae) fed fish had higher levels of glutathione, SOD, glutathione peroxidase, and CAT factors, but lower levels of malondialdehyde, suggesting that fucoidan protects cells from damage [65]. Yang et al. [66] reported increased levels of antioxidant enzymes when fish were administered fucoidan supplementation. A diet containing algae increased antioxidant enzymes such as POD and SOD in rainbow trout [67].

On the other hand, The evaluation of the survival rate of Nile tilapia following exposure to *S. agalactiae* indicated an increase in fish resistance in treatments with algae extract, suggesting that *L. caspica* extract boosts the fish's immune system. Fish survival following exposure to *A. hydrophila* was considerably enhanced when *Ctenopharyngodon idella* was fed with polysaccharide isolated from *Porphyra yezoensis* [68]. In addition, in another study, the impact of *Sarcodia suiae* algae extract on Nile tilapia survival following exposure to *S. agalactiae* was dramatically enhanced [69]. Interestingly, previous investigation on the impact of a live attenuated vaccine against *S. agalactiae* infection in *O. niloticus* demonstrated the survival rate of vaccinated fish was 85%–95%, whereas the control group had a survival rate of 0%–15% [11]. Therefore, our results indicate that *L. caspica* stimulates the immune system similarly to vaccines [70].

## 5. Conclusion

According to our findings, dietary administration of *L. caspica* algae extract had a positive impact on blood, safety, antioxidant, and biochemical indicators in Nile tilapia. Therefore, the administarion of this algae can be considered a promising and simple strategy to deal with bacterial disease in aquaculture since it has a beneficial impact on bacterial infection of *S. agalactiae*, which is one of the most common diseases of Nile tilapia on farms. In addition, it can be used as an alternative to antibiotics and decrease chemical usage in tilapia aquaculture. However, more research on the dosage of *L. caspica*, extraction techniques, and immune genes is required.

## Figures and Tables

**Table 1:** Hematological changes of Nile tilapia fed diets with different levels of *L. caspica*.

Parameters	Day 25				Day 50				Interaction
	Control	0.5%	1%	2%	Control	0.5%	1%	2%	
WBC (10^3^/*µ*l^−1^)	5.50 ± 0.20^aA^	4.90 ± 0.27^aA^	5.30 ± 0.13^aA^	5.80 ± 0.26^aA^	6.80 ± 0.35^aA^	7.00 ± 0.20^aB^	9.10 ± 0.39^bB^	9.00 ± 0.13^cB^	*P* < 0.0001
RBC (10^6^/*µ*l^−1^)	1.76 ± 0.06^aA^	1.72 ± 0.02^aA^	1.74 ± 0.01^aA^	1.76 ± 0.03^aA^	1.81 ± 0.05^aA^	1.78 ± 0.03^aA^	1.93 ± 0.04^aB^	1.91 ± 0.05^aB^	*P* < 0.0295
HB (g/dL)	7.06 ± 0.15^aA^	7.26 ± 0.28^aA^	8.40 ± 0.10^bA^	8.83 ± 0.15^bA^	7.53 ± 0.05^aA^	7.66 ± 0.40^aA^	9.20 ± 0.30^bB^	7.86 ± 0.35^aB^	*P* < 0.0001
HCT%	36.0 ± 1.00^aA^	33.6 ± 2.08^aA^	33.0 ± 1.00^aA^	34.3 ± 1.15^aA^	36.3 ± 2.08^aA^	36.6 ± 1.52^aA^	40.0 ± 1.00 ^aB^	39.6 ± 0.57^aB^	*P* < 0.0046
MCV (FL)	204 ± 4.35^aA^	201 ± 1.00^aA^	199 ± 2.64^aA^	202 ± 3.60^aA^	211.3 ± 3.78^aA^	206.6 ± 1.52^aA^	215 ± 4.35^aB^	211 ± 8.88^aB^	NS (*P* = 0.2285)
MCH (pg)	46.4 ± 2.15^aA^	46.8 ± 1.67^aA^	46.73 ± 1.30^aA^	46.13 ± 0.83^aA^	48.0 ± 0.10^aA^	47.83 ± 1.72^aA^	47.8 ± 0.95^aA^	47.83 ± 1.23^aA^	NS (*P* = 0.9593)
MCHC (g/dL)	21.76 ± 1.53^aA^	21.06 ± 0.75^aA^	20.93 ± 0.87^aA^	20.8 ± 0.88^aA^	22.9 ± 1.11^aA^	21.53 ± 0.35^aA^	23.33 ± 1.34^aA^	23.56 ± 0.96^aA^	NS (*P* = 0.2234)
Neutrophilis (%)	17.3 ± 1.52^aA^	13.0 ± 1.73^aA^	17.0 ± 1.73^aA^	18.3 ± 1.52^aA^	17.33 ± 3.21^aA^	18.0 ± 1.73^aB^	20.6 ± 0.57^aA^	22.6 ± 0.57^aA^	NS (*P* = 0.1032)
Lymphocytes (%)	73.3 ± 2.08^aA^	71.6 ± 0.57^aA^	76.6 ± 1.52^aA^	76.0 ± 2.64^aA^	80.3 ± 1.52^aB^	79.6 ± 0.57^aB^	80.0 ± 1.00^aA^	80.0 ± 2.64^aA^	NS (*P* = 0.0965)
Monocytes (%)	4.30 ± 0.57^aA^	5.00 ± 1.00^aA^	4.60 ± 0.57^aA^	4.3 ± 0.57^aA^	4.60 ± 0.57^aA^	5.60 ± 0.57^aA^	6.30 ± 0.57^aA^	6.30 ± 0.57^aB^	NS (*P* = 0.1200)
Eosinophils (%)	0.60 ± 0.57^aA^	0.30 ± 0.57^aA^	0.60 ± 0.57^aA^	0.60 ± 0.57^aA^	1.30 ± 0.57^aA^	1.30 ± 0.57^aA^	1.60 ± 0.57^aA^	2.30 ± 0.57^aB^	NS (*P* = 0.5162)

Data are provided as mean ± SD (*n* = 9 fish per treatment). Means in identical rows with different letters considerably vary (*P* < 0.05). Means in rows with identical lettering do not vary substantially (*P* > 0.05). RBC, red blood cells; WBC, white blood cells; Ht, hematocrit; Hb, hemoglobin; MCV, mean corpuscular volume; MCH, mean corpuscular hemoglobin; MCHC, mean corpuscular hemoglobin concentration.

**Table 2:** Serum immunological changes of Nile tilapia fed diets with different levels of *L. caspica*.

Parameters	Day 25				Day 50				Interaction
	Control	0.5%	1%	2%	Control	0.5%	1%	2%	
Lysozyme (U/min)	18.95 ± 1.23^aA^	21.5 ± 1.12^aA^	21.2 ± 1.06^aA^	21.92 ± 0.22^aA^	20.46 ± 0.45^aA^	25.3 ± 2.68^bB^	25.3 ± 0.93^bB^	27.15 ± 0.20^bB^	NS (*P* = 0.1074)
IgM (mg dL^−1^)	40.23 ± 0.25^aA^	42.3 ± 0.98^aA^	45.5 ± 1.32^bA^	47.4 ± 0.51^bA^	41.2 ± 1.10^aA^	53 ± 0.50^bB^	54.3 ± 0.57^bB^	59 ± 1.32^bB^	*P* < 0.0001
C3 (mg dL^−1^)	39.6 ± 0.57^aA^	41.6 ± 0.65^aA^	47.06 ± 1.00^bA^	48.96 ± 1.00^bA^	40.5 ± 0.5^aA^	44.6 ± 0.57^bB^	56.1 ± 0.85^bB^	59.7 ± 0.60^bB^	*P* < 0.0001

Data are provided as mean ± SD (*n* = 9 fish per treatment). Means in identical rows with different letters considerably vary (*P* < 0.05). Means in rows with identical lettering do not vary substantially (*P* > 0.05). Significantly different (*P* < 0.05) are the main effects of time on the means of rows with different capital letters. IgM, immunoglobulin M; C3, complement 3.

**Table 3:** Serum biochemical changes of Nile tilapia fed diets with different levels of *L. caspica*.

Parameters	Day 25				Day 50				Interaction
	Control	0.5%	1%	2%	Control	0.5%	1%	2%	
ALP (U/L)	51.0 ± 3.80^aA^	68.75 ± 19.13^aA^	48.0 ± 1.80^aA^	72.25 ± 8.40^aA^	53.65 ± 2.60^aA^	91.25 ± 8.56^bA^	99.6 ± 4.11^bB^	104.25 ± 4.87^bB^	*P* = 0.0012
ALT (U/L)	3.80 ± 0.30^aA^	4.94 ± 0.52^aA^	3.43 ± 0.31^aA^	5.78 ± 1.57^aA^	4.80 ± 0.26^aA^	6.01 ± 0.20^aA^	5.62 ± 0.08^aB^	9.80 ± 1.11^bB^	*P* = 0081
AST (U/L)	240 ± 2.00^aA^	155.6 ± 18.50^aA^	214.67 ± 41.28^aA^	187.3 ± 28.71^aA^	269.3 ± 27.01^aA^	231.3 ± 6.03^aA^	305 ± 122.05^aA^	357 ± 136.52^aA^	NS (*P* = 0.3778)

Data are provided as mean ± SD (*n* = 9 fish per treatment). Different letters in the same rows produce drastically different means. Means in rows with identical lettering do not substantially vary (*P* > 0.05). Significantly different (*P* < 0.05) are the main effects of time on the means of rows with different capital letters. ALT, alanine aminotransferase; AST, aspartate aminotransferase.

**Table 4:** Antioxidant changes of Nile tilapia fed diets with different levels of *L. caspica*.

Parameters	Day 25				Day 50				Interaction
	Control	0.5%	1%	2%	Control	0.5%	1%	2%	
CAT (*µ*m/g)	0.34 ± 0.01^aA^	0.35 ± 0.006^aA^	0.31 ± 0.01^aA^	0.36 ± 0.01^aA^	0.36 ± 0.008^aA^	0.53 ± 0.05^bB^	0.66 ± 0.05^bB^	0.64 ± 0.04^bB^	*P* < 0.0001
POD (*µ*m/g)	1.15 ± 0.16^aA^	1.35 ± 0.04^aA^	2.70 ± 0.07^bA^	1.82 ± 0.09^bA^	1.26 ± 0.08^aA^	1.48 ± 0.19^aA^	2.72 ± 0.15^bA^	2.27 ± 0.15^bB^	NS (*P* = 0.0568)
SOD (U/g)	0.037 ± 0.001^aA^	0.057 ± 0.002^bA^	0.063 ± 0.006^bA^	0.062 ± 0.003^bA^	0.034 ± 0.001^aA^	0.055 ± 0.003^bA^	0.053 ± 0.006^bA^	0.062 ± 0.001^bA^	NS (*P* = 2056)

Data are provided as mean ± SD (*n* = 9 fish per treatment). There is a statistically significant difference (*P* < 0.05), between the means of identical rows denoted by distinct letters. Means in rows with identical lettering do not substantially vary (*P* > 0.05). Significantly different (*P* < 0.05) are the main effects of time on the means of rows with different capital letters. SOD, superoxide dismutase; POD, peroxidase; CAT, catalase.

## Data Availability

All data generated or analyzed during this study are included in this published article.
